# Synthesis and crystal structure of *N*,*N*′-(1,4-phenyl­enedi­methyl­idyne)bis­(2-phenyl­benzenamine)

**DOI:** 10.1107/S2056989025007996

**Published:** 2025-09-11

**Authors:** Siva Kumar Srinivasan, Biraj Jyoti Borah, Vittal Babu Gudimetla

**Affiliations:** a Department of Chemistry School of Basic and Applied Sciences Central University of Tamil Nadu Thiruvarur - 610005 Tamil Nadu India; b Department of Chemical Sciences Tezpur University,Tezpur - 784028 Assam India; Universidade de Sâo Paulo, Brazil

**Keywords:** Single crystal XRD structure, *N*,*N*′-(1,4-phenyl­enedi­methyl­idyne)bis­(2-phenyl­benzenamine), 1,4 di­imine, π–π inter­actions, 1,4-phenyl­enedi­imine, biphenyl substituents, steric hindrance, non-planar conformations

## Abstract

This work reports the synthesis and structural aspects of the title compund, a conjugated 1,4-phenyl­enedi­imine, C_32_H_24_N_2_, which is encumbered with two steric biphenyl moieties. The compound possesses an inversion center and crystallizes in monoclinic space group *P*2_1_/*n*. The presence of biphenyl units across the conjugated di­imine results in a non-planar structure, where the two biphenyl units are twisted away from the plane of the central phenyl ring. The single-crystal XRD (SC-XRD) study of this mol­ecule provides insight into its mol­ecular conformation, bond parameters, and inter­molecular inter­actions, which are essential for understanding its potential as a key building block for the construction of inter­esting supra­molecular structures and fine-tuning their properties.

## Abstract 2

This research highlights the synthesis, characterization and single-crystal XRD structure of a conjugated 1,4-phenyl­enedi­imine encumbered with biphenyl units. The biphenyl units and the central phenyl ring are connected through the imine bonds, creating three aromatic planes that are almost perpendicular to each other. The overall structure of the mol­ecule was found to be controlled by substituent’s steric bulk and the weak inter­actions assisted by the nitro­gen of the imine group.

## 1. Chemical context

Di­imine ligands are characterized by the presence of two C=N π-bonds. The presence of π (carbon–nitro­gen) bonds and the lone pair of electrons on the nitro­gen facilitates an excellent metal coordination ability and they have applications in coordination chemistry, catalysis and supra­molecular chemistry (Belowich & Stoddart, 2012 ▸). The relative position of the first imine bond to the second imine unit in the mol­ecule could result in the diversification of properties, accordingly 1,2-di­imines (α-di­imines) and 1,4-phenylenedi­imines are well known in the literature and their structural aspects are important for the design and development of new mol­ecules. 1,2-Di­imines, often referred as DADs (*i.e.* 1,4-di­aza-1,3-butadienes; Nikolaevskaya *et al.*, 2020 ▸), are a versatile class of chelating nitro­gen-donor ligands, recognized for their ability to stabilize various transition-metal complexes across different oxidation states (Ellandula *et al.*, 2017 ▸; Roy *et al.*, 2011 ▸). The single crystal XRD structures of such 1,2-di­imines have been reported in the literature (Joy *et al.*, 2023 ▸) but such studies of 1,4-phenylenedi­imines are not prevalent. In 1,4-phenylenedi­imines, the two imine groups are separated by a bridging phenyl (ar­yl) unit, which could result in an extended π-conjugated system featuring three perpendicular conjugated aromatic planes, separated by the imine bonds. Thus, it provides an inter­esting rigid structural framework capable of fine tuning the π conjugation across the mol­ecule and its properties (electronic, optical) through the introduction of appropriate substituents (Irfan *et al.*, 2022 ▸). Therefore, understanding the precise structure and conformations of such a conjugated 1,4-phenylenedi­imine in its uncoordinated state will be useful for the rational design and development of new mol­ecules with desired features. The presence of bulky N-substituents (such as biphen­yl) introduces considerable torsional distortion on the extended π-conjugation and its mol­ecular structure, which is an important aspect when making materials for a variety of applications (Kreisel *et al.*, 2008 ▸; Dabb & Fletcher, 2015 ▸; Duchemin *et al.*, 2023 ▸; Liu *et al.*, 2024 ▸; Wu *et al.*, 2023 ▸). In this context, this work reports the studies of newly synthesized *N*,*N*′-(1,4-phenyl­enedi­methyl­idyne)bis­(2-phenyl­benzenamine), and its single crystal XRD structure. The mol­ecule has three distinguishable aromatic planes, where the peripheral biphenyl planes deviate from the plane of the core phenyl ring and thus it exhibits a non-planar twisted geometry, which is controlled and stabilized by the steric influence of the substituent. Mol­ecules exhibiting such structures are found to be useful in the fields of mol­ecular electronics, supra­molecular chemistry and catalysis.
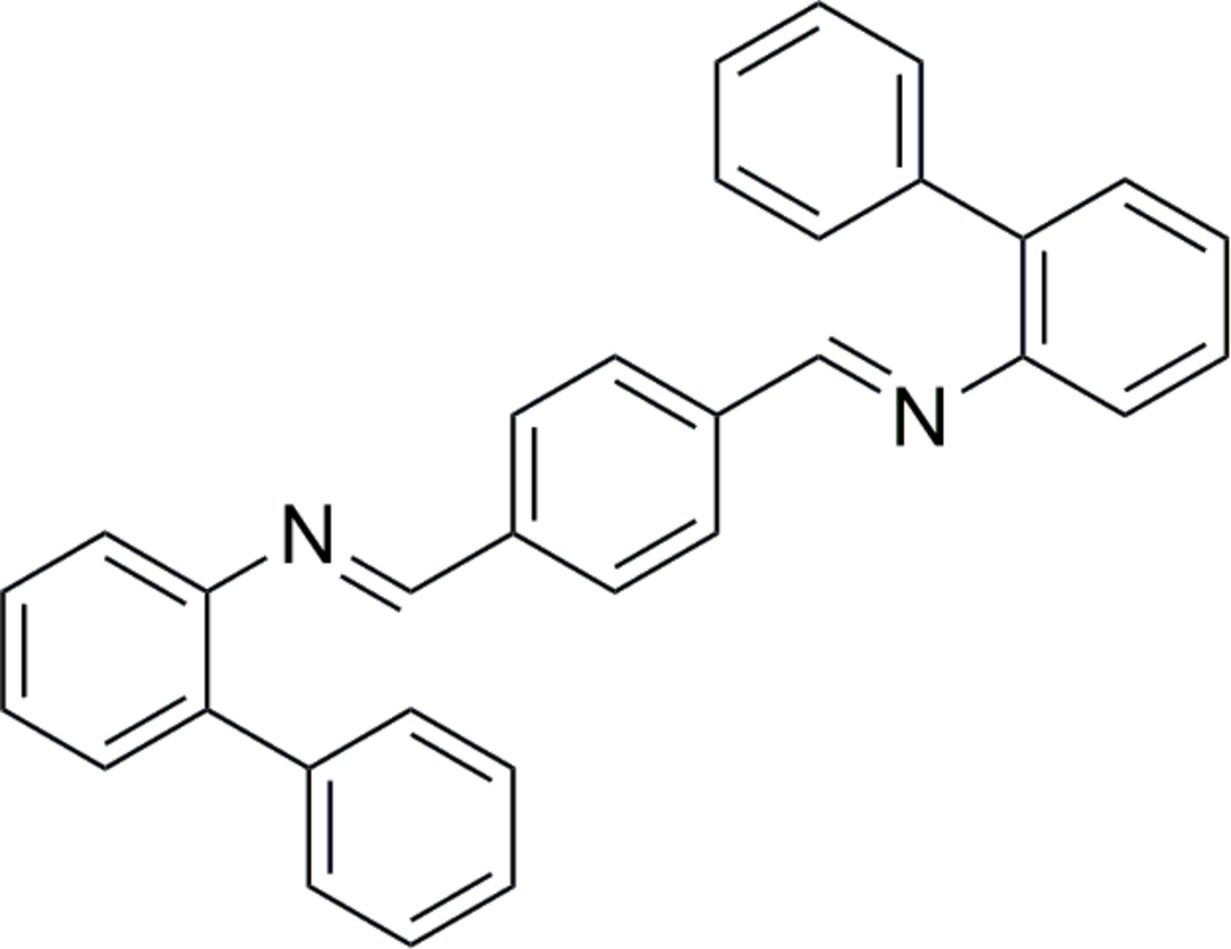


## 2. Structural commentary

**Figure 1:**
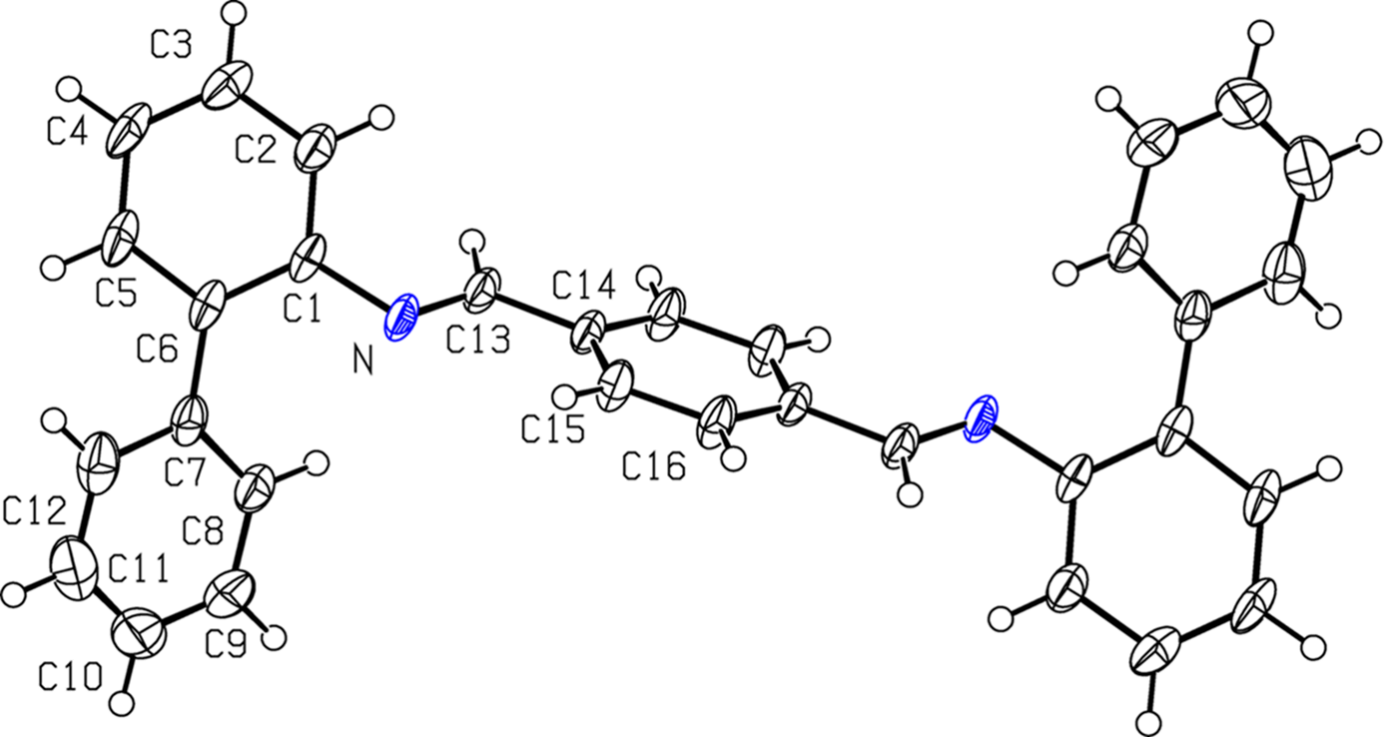
The title compound showing the atom labeling. Displacement ellipsoids are drawn at 50% probability level. Unlabeled atoms are generated by the symmetry operation −*x* + 1, −*y*, −*z* + 1.

The title compound (Fig. 1 ▸) crystallizes in space group *P*2_1_/*n* with two mol­ecules in the unit cell (*Z* = 2), possesses an inversion center and the ligand adopts a non-planar structure in which the two flanking imine bonds are in a *trans*-(*E*,*E*′) configuration. The carbon—nitro­gen C13-N bond distance in the 1,4-phenylenedi­imine unit was found to be 1.278 (2) Å, which is consistent with the literature values. The bond angles involving the imine units, C14—C13—N and C13—N—C1, are 123.83 (13) and 116.95 (12)°, suggesting a slight deviation from pure *sp*^2^ character in the carbon and nitro­gen atoms of the imine bond. The bond length connecting the imine carbon to the central 1,4-phenyl ring [C13—C14 = 1.4695 (19) Å] suggests delocalization of the π-cloud from the phenyl ring and the imine unit. The C14—C13—N—C1 torsion angle between the central phenyl ring and the phenyl moiety of the peripheral biphenyl unit of 170.06 (13)° reiterates that the core phenyl unit and the phenyl moiety of the biphenyl unit are not in same plane, due to the lone pair of electrons on nitro­gen and resulting imine twist. The core 1,4-phenyl­ene ring is almost perpendicular to the outer planes of the biphenyl units as demonstrated by the torsion angles between the core phenyl ring and the imine units [C15—C14—C13—N and C16—C14—C13—N = −15.0 (2) and 167.10 (15)°, respectively]. Similarly, the torsion angles between the imine unit and phenyl moiety of the biphenyl unit, C13—N—C1—C2 and C13—N—C1—C6 are −44.30 (19) and 141.90 (15)°, respectively, suggesting a clear deviation from planarity between the central phenyl and the biphenyl unit connected through the imine bonds. Furthermore, the biphenyl unit itself is twisted [C12—C7—C6—C5 and C1—C6—C7—C8 = −32.9 (2) and −34.6 (2)°, respectively] clearly reflecting that the phenyl rings in the biphenyl are twisted to avoid steric crowding. The carbon–carbon bond lengths in the benzene ring of the biphenyl unit are in the range 1.37–1.40 Å, indicating delocalization of π-electrons. This non-planar arrangement helps to alleviate intra­molecular steric repulsion between the phenyl rings and influences the overall electronic conjugation and conformational flexibility of the ligand. In the solid-state structure, the distance between H8 (*ortho*-proton from the outer phenyl unit of the biphenyl moiety) and N (imine nitro­gen) is 2.52 Å, indicating a weak intra­molecular C—H⋯N inter­action within the mol­ecule, which dictates the orientation of the outer phenyl unit of the biphenyl group. Similarly, N⋯H15 = 2.67 Å.

## 3. Supra­molecular features

**Figure 2:**
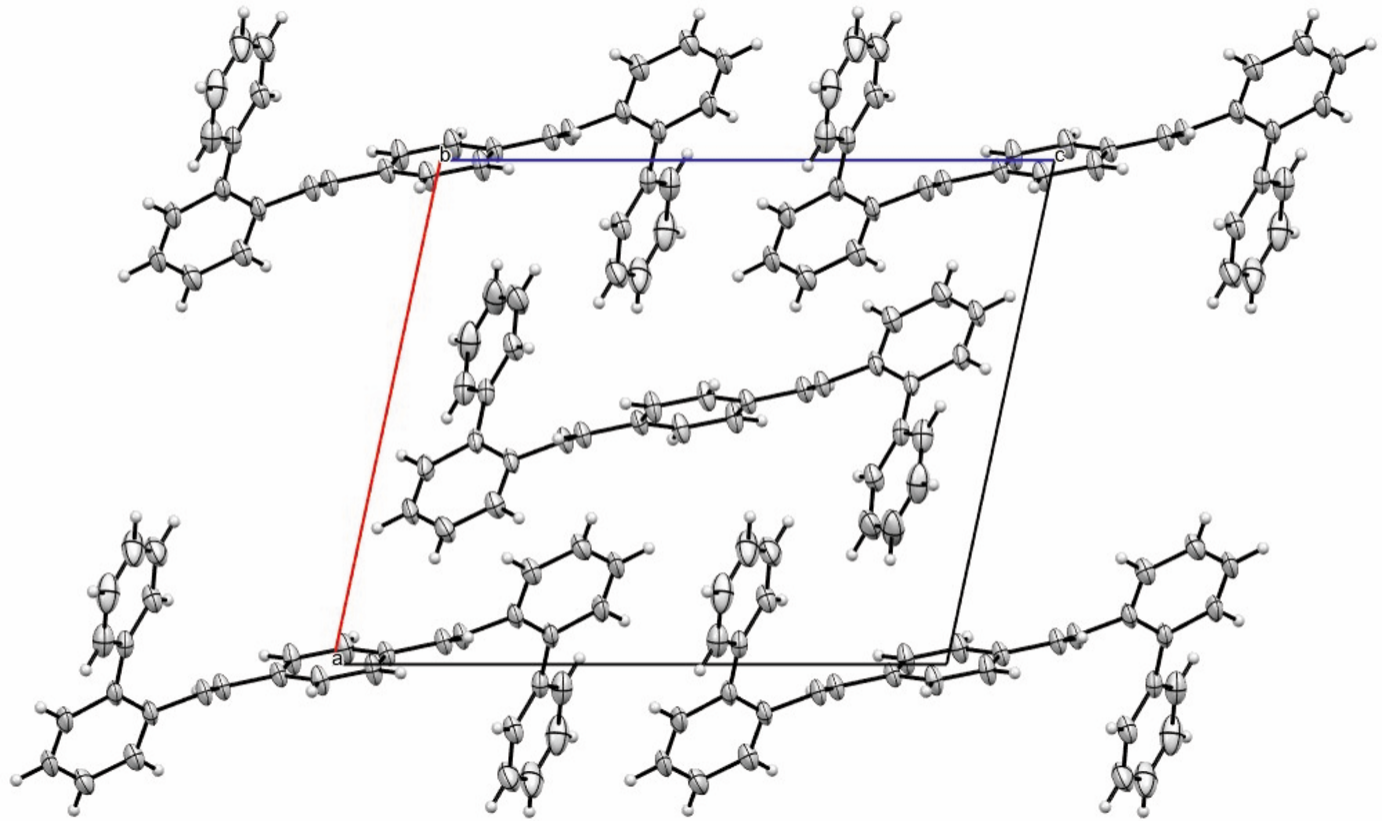
A view along the *b* axis of the crystal packing of the title compound.

In the crystal (Fig. 2 ▸), a single 1,4-phenylenedi­imine unit is surrounded by two other mol­ecules. Moreover, it was observed that the two biphenyl units are placed exactly above the two imine bonds of the 1,4-di­imine. Therefore, in addition to sterics, the nitro­gen from the imine group through its electronegative nature and its inter­actions with the aromatic rings controls the overall structure of the mol­ecule.

## 4. Database survey

A search of the Cambridge Structural Database (webCSD, accessed on August 3, 2025; Groom *et al.*, 2016 ▸) confirmed that the structure of the title compound had not previously been deposited. However, a compound incorporating the 1,4-phenylenedi­imine ligand useful for covalent organic frameworks (COFs) has been reported (WILSIT0; Liu *et al.*, 2024 ▸). Ojala *et al*. (2007 ▸; LICGAG) and Chakraborty *et al.* (2002 ▸; XIGRIO) reported the crystal structures of compounds with methoxy and hydroxy substituents for studying its metal complexation properties. On this basis, some structures with a non-symmetrical 1,2 di­imine ligand have been reported (BICGON and BICGUT; Joy *et al.*, 2023 ▸) and well as a few metal complexes [MUPZOP (Kanno *et al.*, 2020 ▸), JOGPAZ (Sheikh *et al.*, 2019 ▸), NISHOP (Nesterov *et al.*, 2019 ▸), DOJBAI (Roupa *et al.*, 2019 ▸)] have also been explored.

## 5. Synthesis and crystallization

Methanol (25 mL) was taken in a 100 mL round-bottom flask. Terephthalaldehyde (0.250 g, 1.8 mmol, 1.0 equiv.) and 2-amino­biphenyl (0.694 g, 4.1 mmol, 2.2 equiv.) were added and the reaction was allowed to stir at room temperature. Acetic acid (0.07 ml, 1.3 mmol, 0.7 equiv.) was added slowly to this mixture and the reaction was allowed to reflux for 1 h. After completion of the reaction, a light-yellow fluffy precipitate was obtained. It was isolated by vacuum filtration, followed by washing with lesser volume of methanol and the desired product was isolated. This was dissolved in ethyl acetate and hexane (2:1) and X-ray quality crystals were obtained by slow evaporation. Yield: 75% (0.610 g), m.p.: 427–429 K, pale-yellow solid blocks; ^1^H NMR (400 MHz; CDCl_3_): δ 8.461 (*s*, 2H), 7.816 (*s*, 4H), 7.487–7.456 (*m*, 6H), 7.390–7.335 (*m*, 6H), 7.324–7.269 (*m*, 4H), 7.088–7.065 (*dd*, 2H); ^13^C{^1^H} (100 MHz; CDCl_3_): δ (159.5, 149.4, 139.5, 138.8, 135.6, 130.5, 129.1, 128.4, 127.8, 126.9, 126.4, 118.8); FT-IR (Neat, in cm^−1^): 1612 (C=Nstr); HRMS: (ESI^+^): *m*/*z* calculated for [C_32_H_25_N_2_]^+^ ([*M*+H]^+^): 437.20123; found: 437.20544.

## 6. Refinement

Crystal data, data collection and structure refinement details are summarized in Table 1 ▸. H atoms were placed in calculated positions (C—H = 0.95 Å) and refined as riding with *U*_iso_(H) = 1.2*U*_eq_(C).

## Supplementary Material

Crystal structure: contains datablock(s) I. DOI: 10.1107/S2056989025007996/ex2096sup1.cif

Structure factors: contains datablock(s) I. DOI: 10.1107/S2056989025007996/ex2096Isup2.hkl

CCDC reference: 2478745

Additional supporting information:  crystallographic information; 3D view; checkCIF report

## Figures and Tables

**Table 1:** Experimental details

Crystal data	
Chemical formula	C_32_H_24_N_2_
*M*_r_	436.53
Crystal system, space group	Monoclinic, *P*2_1_/*n*
Temperature (K)	296
*a*, *b*, *c* (Å)	12.715 (6), 6.048 (3), 15.121 (7)
β (°)	102.053 (11)
*V* (Å^3^)	1137.1 (9)
*Z*	2
Radiation type	Mo *K*α
μ (mm^−1^)	0.07
Crystal size (mm)	0.32 × 0.28 × 0.26

Data collection	
Diffractometer	Bruker APEXII CCD
Absorption correction	Multi-scan (*SADABS*; Krause *et al.*, 2015 ▸)
*T*_min_, *T*_max_	0.977, 0.981
No. of measured, independent and observed [*I* > 2σ(*I*)] reflections	34178, 2608, 1955
*R*_int_	0.102
(sin θ/λ)_max_ (Å^−1^)	0.650

Refinement	
*R*[*F*^2^ > 2σ(*F*^2^)], *wR*(*F*^2^), *S*	0.045, 0.122, 1.03
No. of reflections	2608
No. of parameters	155
H-atom treatment	H-atom parameters constrained
Δρ_max_, Δρ_min_ (e Å^−3^)	0.19, −0.19

Computer programs: *APEX2* and *SAINT* (Bruker, 2012 ▸), *SHELXS97* (Sheldrick 2008 ▸), *SHELXL* (Sheldrick 2015 ▸) and *SHELXTL* (Sheldrick 2008 ▸).

## supplementary crystallographic information

### N,N'-(1,4-Phenylenedimethylidyne)bis(2-phenylbenzenamine) Crystal data

**Table:**

C_32_H_24_N_2_	*F*(000) = 460
*M**_r_* = 436.53	*D*_x_ = 1.275 Mg m^−^^3^
Monoclinic, *P*2_1_/*n*	Mo *K*α radiation, λ = 0.71073 Å
*a* = 12.715 (6) Å	Cell parameters from 4971 reflections
*b* = 6.048 (3) Å	θ = 2.8–26.4°
*c* = 15.121 (7) Å	µ = 0.07 mm^−^^1^
β = 102.053 (11)°	*T* = 296 K
*V* = 1137.1 (9) Å^3^	Block, colourless
*Z* = 2	0.32 × 0.28 × 0.26 mm

### N,N'-(1,4-Phenylenedimethylidyne)bis(2-phenylbenzenamine) Data collection

**Table:**

Bruker APEXII CCD diffractometer	2608 independent reflections
Radiation source: fine-focus sealed tube	1955 reflections with *I* > 2σ(*I*)
Detector resolution: 2.09 pixels mm^-1^	*R*_int_ = 0.102
phi and ω scans	θ_max_ = 27.5°, θ_min_ = 1.9°
Absorption correction: multi-scan (SADABS; Krause *et al.*, 2015)	*h* = −16→16
*T*_min_ = 0.977, *T*_max_ = 0.981	*k* = −7→7
34178 measured reflections	*l* = −19→19

### N,N'-(1,4-Phenylenedimethylidyne)bis(2-phenylbenzenamine) Refinement

**Table:**

Refinement on *F*^2^	Hydrogen site location: inferred from neighbouring sites
Least-squares matrix: full	H-atom parameters constrained
*R*[*F*^2^ > 2σ(*F*^2^)] = 0.045	*w* = 1/[σ^2^(*F*_o_^2^) + (0.0378*P*)^2^ + 0.3769*P*] where *P* = (*F*_o_^2^ + 2*F*_c_^2^)/3
*wR*(*F*^2^) = 0.122	(Δ/σ)_max_ < 0.001
*S* = 1.03	Δρ_max_ = 0.19 e Å^−^^3^
2608 reflections	Δρ_min_ = −0.19 e Å^−^^3^
155 parameters	Extinction correction: SHELXL2017/1 (Sheldrick 2017), Fc^*^=kFc[1+0.001xFc^2^λ^3^/sin(2θ)]^-1/4^
0 restraints	Extinction coefficient: 0.031 (4)

### N,N'-(1,4-Phenylenedimethylidyne)bis(2-phenylbenzenamine) Special details

**Table:**

Geometry. X-ray crystallography: X-ray reflections were collected on a Bruker APEX-II, CCD diffractometer using Mo Kα (λ = 0.71073 Å) radiation. Data reduction was performed using Bruker SAINT Software (Madison and WI 2008). Intensities for absorption were corrected using SADABS. Structures were solved and refined using SHELXL-2014 with anisotropic displacement parameters for non-H atoms. Hydrogen atom on O was experimentally located in the crystal structure. All C–H atoms were fixed geometrically using the HFIX command in SHELX-TL(Madison and WI 2008). A check of the final CIF file using PLATON did not show any missed symmetry (Spek, 2003). All esds (except the esd in the dihedral angle between two l.s. planes) are estimated using the full covariance matrix. The cell esds are taken into account individually in the estimation of esds in distances, angles and torsion angles; correlations between esds in cell parameters are only used when they are defined by crystal symmetry. An approximate (isotropic) treatment of cell esds is used for estimating esds involving l.s. planes.

### N,N'-(1,4-Phenylenedimethylidyne)bis(2-phenylbenzenamine) Fractional atomic coordinates and isotropic or equivalent isotropic displacement parameters (Å^2^)

**Table:**

	*x*	*y*	*z*	*U*_iso_*/*U*_eq_	
N	0.55575 (10)	0.3106 (2)	0.30035 (8)	0.0324 (3)	
C1	0.59617 (12)	0.3354 (2)	0.21965 (9)	0.0317 (3)	
C2	0.68394 (13)	0.2060 (3)	0.20916 (10)	0.0386 (4)	
H2	0.710192	0.099062	0.252315	0.046*	
C3	0.73276 (14)	0.2333 (3)	0.13607 (11)	0.0442 (4)	
H3	0.789573	0.142434	0.129367	0.053*	
C4	0.69658 (13)	0.3955 (3)	0.07367 (10)	0.0447 (4)	
H4	0.730081	0.418254	0.025322	0.054*	
C5	0.61027 (13)	0.5246 (3)	0.08314 (10)	0.0400 (4)	
H5	0.586139	0.632774	0.039961	0.048*	
C6	0.55710 (12)	0.4996 (2)	0.15533 (9)	0.0318 (3)	
C7	0.46026 (12)	0.6368 (2)	0.15520 (9)	0.0333 (3)	
C8	0.36998 (13)	0.5538 (3)	0.18336 (10)	0.0389 (4)	
H8	0.372829	0.413732	0.209176	0.047*	
C9	0.27648 (15)	0.6767 (3)	0.17343 (12)	0.0524 (5)	
H9	0.217090	0.618317	0.192194	0.063*	
C10	0.27083 (18)	0.8851 (3)	0.13588 (13)	0.0613 (6)	
H10	0.207597	0.966694	0.128621	0.074*	
C11	0.35927 (19)	0.9718 (3)	0.10922 (12)	0.0588 (6)	
H11	0.356032	1.113271	0.084710	0.071*	
C12	0.45256 (16)	0.8505 (3)	0.11857 (11)	0.0456 (4)	
H12	0.511647	0.911609	0.100219	0.055*	
C13	0.54503 (12)	0.1133 (2)	0.32742 (9)	0.0327 (3)	
H13	0.552940	−0.002594	0.288935	0.039*	
C14	0.52092 (11)	0.0590 (2)	0.41581 (9)	0.0302 (3)	
C15	0.53198 (13)	0.2135 (2)	0.48511 (10)	0.0363 (4)	
H15	0.553911	0.356721	0.475664	0.044*	
C16	0.51054 (14)	0.1555 (2)	0.56821 (10)	0.0378 (4)	
H16	0.517224	0.260946	0.613828	0.045*	

### N,N'-(1,4-Phenylenedimethylidyne)bis(2-phenylbenzenamine) Atomic displacement parameters (Å^2^)

**Table:**

	*U*^11^	*U*^22^	*U*^33^	*U*^12^	*U*^13^	*U*^23^
N	0.0422 (7)	0.0385 (7)	0.0210 (6)	−0.0011 (5)	0.0169 (5)	0.0044 (5)
C1	0.0398 (8)	0.0388 (8)	0.0207 (7)	−0.0070 (6)	0.0162 (6)	−0.0001 (6)
C2	0.0449 (9)	0.0452 (9)	0.0302 (8)	−0.0008 (7)	0.0182 (7)	0.0028 (6)
C3	0.0448 (9)	0.0591 (10)	0.0347 (9)	−0.0018 (8)	0.0220 (7)	−0.0055 (8)
C4	0.0456 (9)	0.0710 (11)	0.0230 (8)	−0.0091 (8)	0.0194 (7)	−0.0017 (7)
C5	0.0454 (9)	0.0566 (10)	0.0207 (7)	−0.0097 (7)	0.0130 (6)	0.0059 (7)
C6	0.0390 (8)	0.0405 (8)	0.0185 (7)	−0.0098 (6)	0.0123 (6)	−0.0007 (6)
C7	0.0473 (9)	0.0359 (8)	0.0182 (7)	−0.0036 (6)	0.0105 (6)	−0.0007 (6)
C8	0.0502 (9)	0.0423 (8)	0.0293 (8)	0.0030 (7)	0.0197 (7)	0.0040 (6)
C9	0.0568 (11)	0.0678 (12)	0.0389 (10)	0.0144 (9)	0.0247 (8)	0.0024 (8)
C10	0.0840 (15)	0.0648 (12)	0.0390 (10)	0.0336 (11)	0.0216 (10)	0.0005 (9)
C11	0.1026 (17)	0.0381 (9)	0.0368 (10)	0.0138 (10)	0.0173 (10)	0.0013 (7)
C12	0.0715 (12)	0.0381 (8)	0.0290 (8)	−0.0080 (8)	0.0142 (8)	−0.0004 (7)
C13	0.0424 (8)	0.0378 (8)	0.0224 (7)	0.0000 (6)	0.0168 (6)	0.0012 (6)
C14	0.0387 (8)	0.0340 (7)	0.0223 (7)	0.0009 (6)	0.0164 (6)	0.0033 (5)
C15	0.0557 (10)	0.0302 (7)	0.0288 (8)	−0.0044 (7)	0.0224 (7)	0.0019 (6)
C16	0.0609 (10)	0.0337 (8)	0.0248 (8)	−0.0048 (7)	0.0226 (7)	−0.0035 (6)

### N,N'-(1,4-Phenylenedimethylidyne)bis(2-phenylbenzenamine) Geometric parameters (Å, º)

**Table:**

N—C13	1.2779 (19)	C8—H8	0.9300
N—C1	1.4275 (18)	C9—C10	1.378 (3)
C1—C2	1.399 (2)	C9—H9	0.9300
C1—C6	1.406 (2)	C10—C11	1.375 (3)
C2—C3	1.386 (2)	C10—H10	0.9300
C2—H2	0.9300	C11—C12	1.377 (3)
C3—C4	1.372 (2)	C11—H11	0.9300
C3—H3	0.9300	C12—H12	0.9300
C4—C5	1.378 (2)	C13—C14	1.4695 (19)
C4—H4	0.9300	C13—H13	0.9300
C5—C6	1.407 (2)	C14—C15	1.389 (2)
C5—H5	0.9300	C14—C16^i^	1.393 (2)
C6—C7	1.484 (2)	C15—C16	1.385 (2)
C7—C8	1.398 (2)	C15—H15	0.9300
C7—C12	1.402 (2)	C16—H16	0.9300
C8—C9	1.383 (2)		
			
C13—N—C1	116.95 (12)	C10—C9—C8	120.39 (18)
C2—C1—C6	119.45 (13)	C10—C9—H9	119.8
C2—C1—N	118.46 (13)	C8—C9—H9	119.8
C6—C1—N	121.81 (13)	C11—C10—C9	119.59 (18)
C3—C2—C1	121.65 (15)	C11—C10—H10	120.2
C3—C2—H2	119.2	C9—C10—H10	120.2
C1—C2—H2	119.2	C10—C11—C12	120.45 (18)
C4—C3—C2	119.46 (16)	C10—C11—H11	119.8
C4—C3—H3	120.3	C12—C11—H11	119.8
C2—C3—H3	120.3	C11—C12—C7	121.30 (18)
C3—C4—C5	119.55 (15)	C11—C12—H12	119.4
C3—C4—H4	120.2	C7—C12—H12	119.4
C5—C4—H4	120.2	N—C13—C14	123.83 (13)
C4—C5—C6	122.81 (15)	N—C13—H13	118.1
C4—C5—H5	118.6	C14—C13—H13	118.1
C6—C5—H5	118.6	C15—C14—C16^i^	118.88 (13)
C1—C6—C5	117.05 (14)	C15—C14—C13	121.87 (13)
C1—C6—C7	124.67 (12)	C16^i^—C14—C13	119.21 (13)
C5—C6—C7	118.15 (13)	C16—C15—C14	120.39 (14)
C8—C7—C12	117.22 (15)	C16—C15—H15	119.8
C8—C7—C6	122.18 (14)	C14—C15—H15	119.8
C12—C7—C6	120.36 (14)	C15—C16—C14^i^	120.72 (14)
C9—C8—C7	121.03 (16)	C15—C16—H16	119.6
C9—C8—H8	119.5	C14^i^—C16—H16	119.6
C7—C8—H8	119.5		
			
C13—N—C1—C2	−44.30 (19)	C5—C6—C7—C12	−32.9 (2)
C13—N—C1—C6	141.90 (15)	C12—C7—C8—C9	1.4 (2)
C6—C1—C2—C3	−0.9 (2)	C6—C7—C8—C9	−172.90 (15)
N—C1—C2—C3	−174.86 (14)	C7—C8—C9—C10	−0.4 (3)
C1—C2—C3—C4	2.0 (2)	C8—C9—C10—C11	−0.8 (3)
C2—C3—C4—C5	−1.9 (2)	C9—C10—C11—C12	1.0 (3)
C3—C4—C5—C6	0.7 (2)	C10—C11—C12—C7	0.0 (3)
C2—C1—C6—C5	−0.3 (2)	C8—C7—C12—C11	−1.2 (2)
N—C1—C6—C5	173.44 (13)	C6—C7—C12—C11	173.21 (15)
C2—C1—C6—C7	175.54 (14)	C1—N—C13—C14	170.06 (13)
N—C1—C6—C7	−10.7 (2)	N—C13—C14—C15	−15.0 (2)
C4—C5—C6—C1	0.4 (2)	N—C13—C14—C16^i^	167.10 (15)
C4—C5—C6—C7	−175.70 (14)	C16^i^—C14—C15—C16	−0.9 (3)
C1—C6—C7—C8	−34.6 (2)	C13—C14—C15—C16	−178.76 (14)
C5—C6—C7—C8	141.19 (15)	C14—C15—C16—C14^i^	0.9 (3)
C1—C6—C7—C12	151.32 (15)		

Symmetry code: (i) −*x*+1, −*y*, −*z*+1.
